# Using a Novel Wireless-Networked Decentralized Control Scheme under Unpredictable Environmental Conditions

**DOI:** 10.3390/s151128690

**Published:** 2015-11-12

**Authors:** Chung-Liang Chang, Yi-Ming Huang, Guo-Fong Hong

**Affiliations:** Department of Biomechatronics Engineering, National Pingtung University of Science and Technology, Pingtung County, 91201, Taiwan; E-Mails: snake576@gmail.com (Y.-M.H.); e9744043@yahoo.com.tw (G.-F.H.)

**Keywords:** fuzzy logic inference, graphic user interface, wireless sensor network, environment control

## Abstract

The direction of sunshine or the installation sites of environmental control facilities in the greenhouse result in different temperature and humidity levels in the various zones of the greenhouse, and thus, the production quality of crop is inconsistent. This study proposed a wireless-networked decentralized fuzzy control scheme to regulate the environmental parameters of various culture zones within a greenhouse. The proposed scheme can create different environmental conditions for cultivating different crops in various zones and achieve diversification or standardization of crop production. A star-type wireless sensor network is utilized to communicate with each sensing node, actuator node, and control node in various zones within the greenhouse. The fuzzy rule-based inference system is used to regulate the environmental parameters for temperature and humidity based on real-time data of plant growth response provided by a growth stage selector. The growth stage selector defines the control ranges of temperature and humidity of the various culture zones according to the leaf area of the plant, the number of leaves, and the cumulative amount of light. The experimental results show that the proposed scheme is stable and robust and provides basis for future greenhouse applications.

## 1. Introduction

The benefit of using a greenhouse for plant cultivation is that it can reduce damaged crops caused by pests, decrease microbial contamination and increase crop quality and quantity [1,2,3]. Nevertheless, a greenhouse is a semiclosed thermodynamic system, which is influenced by the outside air temperature, humidity, solar radiation intensity, wind speed, and CO_2_ concentration, as well as the indoor heating system, lighting, and heat dissipation of other equipment, crop soil heat dissipation, and moisture diffusion variables, all of which means the environment in the system is prone to change. Therefore, the cultivators must use advanced facilities to regulate heat exchange between the greenhouse and external environment manually or automatically, so that the environmental conditions inside the greenhouse can maintain plant growth. Common greenhouse environmental control technologies preset the upper and lower limit values of the environmental control parameters, as provided by various environmental control facilities according to the user’s experience, which then increase or reduce the temperature and humidity.

However, the greenhouse environmental control facilities, such as shade nets, wetted pads, spray facilities, and blowers, are installed in a specific position in the greenhouse to implement environmental control. This practice, for a large greenhouse, often results in non-uniform temperature, humidity, and CO_2_ value distribution in various zones inside the overall greenhouse. As each position is at a different solar angle, this results in different illumination intensities in the various zones of the greenhouse, meaning the plants in the greenhouse receive non-uniform light, which will certainly cause differences in the growth of plants in various zones.

To standardize quality of products, an environmental condition regulation method that creates multiple zones in the greenhouse is required. The most suitable environmental condition is created based on the plant growth response and conditions of different zone in the greenhouse. This method can prevent the above-mentioned problem and reduce energy consumption to achieve standardized production. In addition, the early greenhouse environmental control system was huge and wired, thus, increasing the wire acquisition cost and difficulties in construction maintenance. In recent years, many greenhouse practitioners have set up wireless networks in greenhouses to solve the above problems. This method enables the users to know the running conditions of various facilities in the greenhouse and detect changes in the environmental factors in the greenhouse from a notebook, a personal computer (PC), or mobile devices, and they can even operate various environmental control facilities via a remote interface, in order to implement the expected environmental conditions in the greenhouse. At present, the common communication transmission standards on the market include Bluetooth, WiFi, and Zigbee, *etc*. Embedded wireless sensor modules are accepted by many users for their low energy consumption, compactness, and low cost [4,5,6,7,8,9,10,11]. Besides, control facilities and monitoring devices are developing towards microminiaturization, intelligence, and unified specifications [12,13,14,15]. However, as the climatic factors of greenhouses have strong relationships with each other, it is difficult to build an environmental climate model. Thus, performance when using different environment control methods often depends on the precision of that climate model for the greenhouse. Many simplified models and intelligent control method are used for greenhouse application, such as neural networks [16], model predictive control [17], gradient modeling [18], dynamic modeling [19], wireless-based relay control [20], machine learning [21], and multi-stage fuzzy logic control [22]. However, different zones in the greenhouse present different environmental parameters due to different angle of arrival for the sunlight outside the greenhouse. Therefore, this study proposes a wireless-networked decentralized fuzzy control scheme to accurately regulate the environmental parameters in the various zones of a greenhouse.

The proposed fuzzy rule-based inference method has been applied to engineering applications. The purpose is to solve ill-defined control problems. As long as the designer effectively designs the database, the uncertainty problem can be effectively solved [23]. Through many methods have currently been utilized to compensate for the drawbacks of a fuzzy logic controller (FLC), such as supervised learning [24,25] or reinforcement learning [26,27], the structure of these methods is more complex and a large amount of computation time is required during the execution process in order to adjust FLC parameters [28]. In terms of hardware implementation and real-time computation, they are not as easy as FLC.

In addition, the wireless sensor/actuator network technique is employed to reduce the cost of wire layout in the greenhouse and enhance performance of greenhouse management [29,30]. In comparison to the traditional available control system [23,31,32], the proposed system has the following characteristics:

- The high stability and robust micro-electromechanical (MEM) sensing and control devices are used to regulate and control the environmental parameters inside the greenhouse;
- Remote monitoring displays current temperature and humidity changes in the various cultivation zones in the greenhouse;
- The control range of temperature and humidity are adjusted automatically based on different plant growth stages to achieve plant growth optimization;
- Multi-zone decentralized control based on fuzzy rule-based inference for planting standardization and diversification;
- A wireless sensing network is utilized, which reduces the cost of wire mounting between sensing nodes, actuator nodes, and control nodes and provides high mobility, cultivation data collection and processing, and good control flexibility.

The organization of this study is as follows: Section 2 depicts the proposed scheme, including the design concept of wireless greenhouse monitoring and automation and design process of decentralized fuzzy control scheme. Section 3 elaborates the practice implementation of hardware and software and system testing for the proposed scheme, such as image processing and data reception and measurement. The performance analysis of the proposed system is illustrated in Section 4. The experiment results and discussion are also given in this section. The last section is the conclusion, which offers recommendations for additional study and investigation.

## 2. Methodology

This study proposed a wireless-networked decentralized fuzzy control scheme to create different temperature and humidity conditions in a greenhouse, which results in planting standardization and diversification. The design and implementation process of the proposed scheme is illustrated in this section.

### 2.1. Design Concept

**Figure 1:**
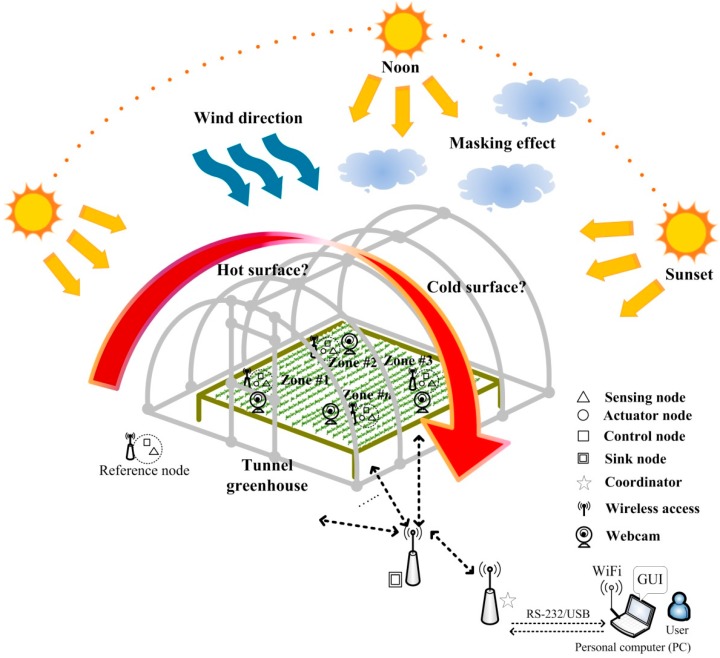
The proposed system architecture is under unpredictable environmental conditions.

Although the early environmental control method can effectively adjust the environment parameters in the greenhouse [31,32], the lighting intensity at different positions in the greenhouse differs due to a different emission angle for the sun. This factor affects the temperature and humidity of one specific position. Besides, the sunlight blanking degree, such as from a cloud or the building, and wind direction all influence the environmental parameters of each position in the greenhouse. These problems of difference cannot be solved by the current controlled-environment facility technique. Thus, this study proposes one decentralized fuzzy control scheme in combination with one highly-stable and robust star-type wireless sensor network technique. The whole system structure is shown in Figure 1.

There is a total of *N* cultivation zones in the greenhouse. Each zone includes a sensing node, control a node and an actuator node. The temperature and humidity sensors, a light sensor, a CO_2_ sensor, a heater, a humidifier, and a fan are placed in each zone of the greenhouse. The micro-controller in the sink node transfers the environmental data collected from sensors in each zone via Zigbee transmission interface to the coordinator. The total data is then sent to the PC via RS-232 or universal serial bus (USB). The graphic user interface (GUI) displays the environmental data of various zones and provide the manual mode and automatic mode options for user to manage and control the environment parameters in Zone #*n* (*n* = 1, 2, 3*…*, *N*). The temperature, humidity, CO_2_, photo flux values, and plant growth response can be automatically recorded, and the user can observe the data in real-time. The decentralized control system is performed and to regulate the environmental factors in each zone of the greenhouse under the automatic mode.

### 2.2. Decentralized Fuzzy Control Scheme

**Figure 2:**
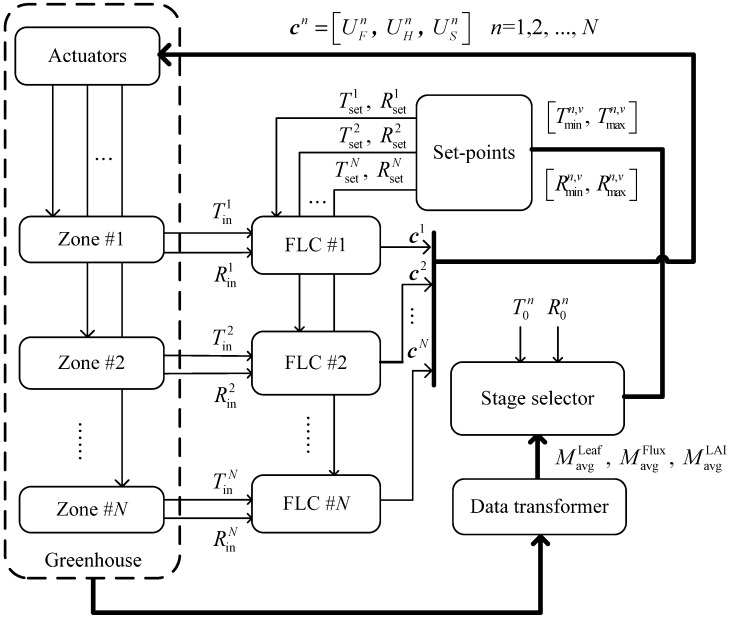
The block diagram of decentralized fuzzy control scheme.

The proposed scheme consists of N fuzzy logic controllers (FLCs), a stage selector, a data a transformer, an actuator module, and a set-points module (see Figure 2). The micro-environmental sensing data of various zones are sent to the fuzzy logic controllers, where fuzzy rule-based inference in the controller decides the starting conditions of the actuators in various zones and controls the actuator output. The v value of the plant growth stage is determined by the average leaf area index ($M_{\text{avg}}^{\text{LAI}}$), the number of leaves ($M_{\text{avg}}^{\text{Leaf}}$), and the cumulative amount of light ($M_{\text{avg}}^{\text{Flux}}$). When the v value is determined, the selector exports a temperature maximum ($T_{\text{max}}^{n,v}$), temperature minimum ($T_{\text{min}}^{n,v}$), humidity maximum ($R_{\text{max}}^{n,v}$), and humidity minimum ($R_{\text{min}}^{n,v}$) to the set-points module for No. n cultivation zone. The selector selects the target temperature and humidity within the range of temperature and humidity randomly. These data will be imported into the FLCs as the target temperature and humidity. The functions of each block are introduced, as follows.

#### 2.2.1. FLC

Fuzzy logic inference technology is usually used to solve unknown or nonlinear control problems in a dynamic system. The core of the technology is to use the fuzzy rules created by expertise to control the system parameters. It is unnecessary to combine the mathematical model for environmental condition changes in the greenhouse with fuzzy controllers for temperature and humidity control. However, it is crucial to know the correlation between the input and output variables of the system, and to study the actuation degree of system output to equipment operation, such as the input voltage of heater (H), pad-fan (S) and the fan (F). The design process of fuzzy logic controller is shown as follows [33]: (a) define the input and output variables; (b) define the membership type of fuzzification; (c) design control rules and fuzzy inference; (d) decision logic; (e) select the defuzzification method.

**Figure 3:**
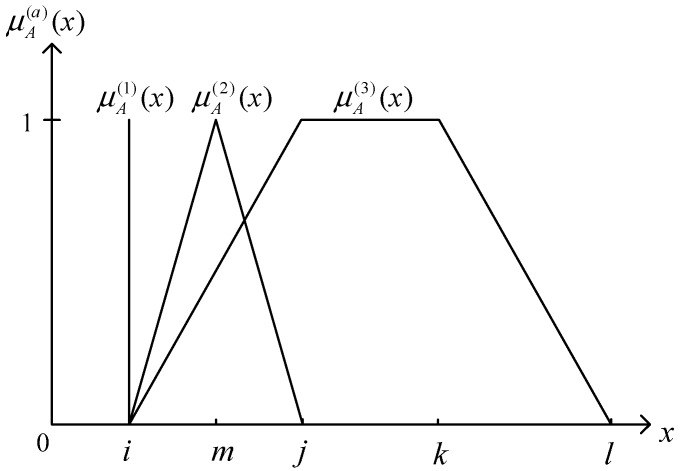
Different types of membership functions.

The first step is to fuzzify the input variables. The selection of input variable directly affects the manifestation of the controlled object, meaning it is necessary to consider the operational conditions that must be observed and measured. In each of fuzzy logic controllers, there are two input variables, which are temperature deviation $T_{e}^{n}$ and humidity deviation $R_{e}^{n}$, defined as follows:

(1) $$T_{e}^{n}=T_{\text{set}}^{n}-T_{\text{in}}^{n}$$

(2) $$R_{e}^{n}=R_{\text{set}}^{n}-R_{\text{in}}^{n}$$

where $T_{\text{set}}^{n}$ and $R_{\text{set}}^{n}$ represent the target temperature and humidity set by controller n, where $T_{\text{in}}^{n}$ and $R_{\text{in}}^{n}$ represent current temperature and humidity, respectively. The fuzzy controller must make decisions according to the difference between the expected variable value and the actual variable value. It is noteworthy that there is mutual coupling correlation between temperature and humidity. Therefore, the temperature and humidity deviation are imported into the $\eta_{1}$ and $\eta_{2}$ factors, and the output equations are described by Equations (3) and (4):

(3) $${\bar{T}}_{e}^{n}=\left(1-\eta_{1}\right)T_{e}^{n}+\eta_{2}R_{e}^{n}$$

(4) $${\bar{R}}_{e}^{n}=\left(1-\eta_{2}\right)R_{e}^{n}+\eta_{1}T_{e}^{n}$$

where $\eta_{1}$ and $\eta_{2}$ values are 0~1. When $\eta_{1}$ and $\eta_{2}$ values are 0, the mutual coupling between temperature and humidity deviation is disregarded. When the $\eta_{1}$ and $\eta_{2}$ values are increased, the fluctuation of temperature and humidity deviation is reduced. Afterwards, the ${\bar{T}}_{e}^{n}$ and ${\bar{R}}_{e}^{n}$ need to be converted into a fuzzy linguistic (fuzzy set) for expression. By determining the number of fuzzy sets used based on the complexity of the control problem in terms of each input or output variable, each variable is matched with 3 different fuzzy sets in each fuzzy logic controller according to its category. Basically, a membership function is utilized to define how much of the temperature (or humidity) deviation “crisp” value is “small (S)”, how much is “moderate (M)”, and how much is “large (B)”. In addition, output variables of each controllers include normalized voltage of fan ($u_{F}^{n}$), voltage of heater ($u_{H}^{n}$), and voltage of pad-fan ($u_{S}^{n}$), whose fuzzy sets are low (L), moderate (M), and high (H), respectively. The membership function for fuzzy set A on the universe of discourse X is defined as $\mu_{A}(x)$: X→[0,1], where each element of X is mapped to a value between 0 and 1. This value, called the degree of membership, quantifies the grade of membership of the element in X to fuzzy set A. The shape of the membership function will influence the degree value of each membership function corresponding to each input value, and directly influence the performance of the controller. The membership functions are defined as singleton membership function ($\mu_{A}^{\left(1\right)}(x)$), triangle membership function ($\mu_{A}^{\left(2\right)}(x)$), and trapezoid membership function ($\mu_{A}^{\left(3\right)}(x)$), respectively, as these membership functions are simple. The different types of membership are shown in Figure 3.

The mathematical equations are expressed as Equations (5)–(7).

Type I: Singleton membership function:

(5) $$\mu_{A}^{\left(1\right)}(x)=\{\begin{array}{ll} 1 & x=i\\ 0 & x\neq i \end{array}$$

The points of the base can be depicted as {s}.

Type II: Triangle membership function: defined by a lower limit i, an upper limit j, and a value *m*, where *i* < *m* < *j*.

(6) $$\mu_{A}^{\left(2\right)}(x)=\{\begin{array}{c} 0,\;x\leq i\;\\ \frac{x-i}{k-i},\;i<x\leq m\;\\ \frac{j-x}{j-k},\;m<x\leq j\;\\ 0,\;x\geq j\; \end{array}$$

The points of base can be demonstrated as [i, m, j].

Type III: Trapezoid membership function: defined by a lower limit i, an upper limit l, a lower support limit j, and an upper support limit k, where *i* < *j* < *k* < *l*.

(7) $$\mu_{A}^{\left(3\right)}(x)=\{\begin{array}{c} 0,\;(x<i)\text{ or }(x>l)\;\\ \frac{x-i}{j-i},\;a\leq x\leq b\;\\ 1,\;j\leq x\leq k\;\\ \frac{l-x}{l-k},\;k\leq x\leq l\; \end{array}$$

The brackets [i, j, k, l] show the points of the base. The type and quantity of membership functions and the input variable range designed by each controller in this system are as listed in Table 1.

**Table 1:** Fuzzification parameter settings and different type of membership function determination.

Variables	Fuzzy Subset (Linguistic Labels)	Type of Membership Function	Points of Base	Range
Temperature deviation (${\bar{T}}_{e}^{n}$)	S	Trapezoid ($\mu_{A}^{\left(3\right)}(x)$)	[−5, −5, −2.5, −0.5]	[−5, 5]
	M	Triangle ($\mu_{A}^{\left(2\right)}(x)$)	[−1.5, 0, 1.5]	
	B	Trapezoid ($\mu_{A}^{\left(3\right)}(x)$)	[0.5, 2.5, 5, 5]	
Humidity deviation (${\bar{R}}_{e}^{n}$)	S	Trapezoid ($\mu_{A}^{\left(3\right)}(x)$)	[−10, −10, −5, −3]	[−10, 10]
	M	Triangle ($\mu_{A}^{\left(2\right)}(x)$)	[−5, 0, 5]	
	B	Trapezoid ($\mu_{A}^{\left(3\right)}(x)$)	[3, 5, 10, 10]	
Fan ($u_{F}^{n}$)	L	Singleton ($\mu_{A}^{\left(1\right)}(x)$)	{0.3}	[0, 1]
	M		{0.6}	
	H		{0.9}	
Heater ($u_{H}^{n}$)	L	Singleton ($\mu_{A}^{\left(1\right)}(x)$)	{0.3}	[0, 1]
	M		{0.6}	
	H		{0.9}	
Pad-fan ($u_{S}^{n}$)	L	Singleton ($\mu_{A}^{\left(1\right)}(x)$)	{0.3}	[0, 1]
	M		{0.6}	
	H		{0.9}	

When the input variables are fuzzified, a group of fuzzy output values shall be obtained by the fuzzy inference process, and this inference process often uses an “If-Then” rule. Table 2 describes the association rule base of input variables and output variables. For example, the fuzzy rule (1) statement in Table 2 is:

(8) $$\text{Rule }(1):\text{ If }\{({\bar{T}}_{e}^{n}\text{ is}S)\;\&\;({\bar{R}}_{e}^{n}\text{ is S})\}\text{ Then }\{(u_{F}^{n}\text{ is}L)\}\;\&\;\{(u_{H}^{n}\text{ is}L)\}\;\&\;\{(u_{S}^{n}\text{ is}L)\}$$

**Table 2:** Fuzzy rules.

${\bar{u}}_{F}^{n},\;{\bar{u}}_{H}^{n},\;{\bar{u}}_{S}^{n}$		${\bar{T}}_{e}^{n}$		
		S	M	B
${\bar{R}}_{e}^{n}$	S	{L, L L}	{M, M L}	{L, H L}
	M	{L, L M}	{M, M M}	{H, H M}
	B	{L, L H}	{M, M H}	{H, H H}

After completion of the inference process, a decision is required. This group of fuzzy output values uses “min-max” synthesis inference for making decisions [33]. During calculation, “∧” is taken as intersection operator and “∨” as the union operator. The maximum output membership value of the entire inference result is as shown in Equation (9):

(9) $$\begin{array}{l} \mu_{C^{\prime }}(z)=\left[\mu_{{\tilde{A}}_{1}}(\alpha )\wedge \mu_{{\tilde{B}}_{1}}(\beta )\wedge \mu_{{\tilde{C}}_{1}}(\gamma )\right]\vee \left[\mu_{{\tilde{A}}_{2}}(\alpha )\wedge \mu_{{\tilde{B}}_{2}}(\beta )\wedge \mu_{{\tilde{C}}_{2}}(\gamma )\right]\;\vee \cdots \\ \;\left[\mu_{{\tilde{A}}_{K}}(\alpha )\wedge \mu_{{\tilde{B}}_{K}}(\beta )\wedge \mu_{{\tilde{C}}_{K}}(\gamma )\right] \end{array}$$

where α, β, and γ denote input variables; z depicts the output variable; and K is the total quantity of fired fuzzy rules. $\mu_{\tilde{\Delta }}(*)$ is defined as Equations (5)–(7). Finally, a crisp output is obtained by defuzzification of the fuzzy output values after inference, where the fundamental purpose is to reduce the fuzzy membership grade to a single output value. The common methods include center of gravity, center of sum, and weighted average method. In this study, the weighted average method is taken for defuzzification (Mark II, 1994), as shown in the Equation (10).

(10) $$U=\sum_{q=1}^{Q}\mu_{C^{\prime }}(z_{q})\;z_{q}/\sum_{q=1}^{Q}\mu_{C^{\prime }}(z_{q})$$

where Q is the fired rule quantity, $\mu_{C^{\prime }}(z_{q})$ can be obtained through Equation (9), and $z_{j}$ is the degree that the premise of rule j is satisfied. U is the normalized voltage, as a driver is adopted, and the scaling output value will range from 0 to 12 V; as the fuzzy membership function output by the system is singleton, $\mu_{C^{\prime }}(z_{q})$ of Equation (10) can correspond to one weight value $w_{q}$. Therefore, Equation (10) can be rewritten as:

(11) $$U=\frac{\sum_{q=1}^{Q}w_{q}\cdot z_{q}}{\sum_{q=1}^{Q}w_{q}}$$

Finally, the nth fuzzy controller exports output data $c^{n}=\left[U_{F}^{n}\text{, }U_{H}^{n}\text{, }U_{S}^{n}\right]$, where $U_{F}^{n}\text{, }U_{H}^{n}\text{, and }U_{S}^{n}$ denote the crisp voltage output of the fan, heater, and pad-fan, respectively.

#### 2.2.2. Data Transformer

The data transformation processing function calculates the leaf area index (LAI) and the cumulative amount of light of the nth cultivation zone, and the calculated values are sent to the stage selector. The leaf area and number of leaves in various culture areas are obtained using a charge-coupled-device (CCD) to record the plant leaf width above the various cultivation zones, and then the leaf area index is calculated by the image processing method [14]. In general, the maximum LAI for vegetables is around 5, and it is ideal when the LAI remains between 3 and 4 [34].

#### 2.2.3. Growth Stage Selector

Many early environmental control methods use the ideal plant physiological model to determine the optimal environmental control value, which is then imported into the actual greenhouse system [35]. This control mode is inflexible, and cannot dynamically adjust the environmental factor values in the greenhouse. Since 2004, dynamic regulation of environmental factors has received the attention of many scholars, and many methods have been proposed [22,36,37]. In the proposed scheme, the output value obtained by each fuzzy controller in the system will be sent to the stage selector, which decides whether or not to start the actuators for various zones. The selector divides the plant growing process into three stages, which are the seedling stage, growth stage, and harvest stage. The required leaf area, number of leaves, and cumulative amount of light in each growth stage are as shown in Table 3. The present stage of the plants is judged according to the plant growth leaf area average, number of leaves, and the required cumulative amount of light, so the three values must conform to the required conditions of the stage so that the growth stage of plants can be defined; otherwise, the previous growth stage will remain. In addition, if two output values are out of the set range, it is the “other” stage, and the previous growth stage remains. Table 3 takes lettuce as an example. The leaf area range of plant growth in each zone can be captured by the image device in the zone and calculated by binary conversion. In the same way, the number of leaves can be obtained from the image captured by image processing recognition. The lighting intensity can be obtained by the light quantum sensor in each zone. When the corresponding plant growth stage is found in Table 3, the growth stage corresponds to the habitat temperature and humidity of the stage.

**Table 3:** Plant growth stages corresponding to plant growth response intervals.

	Parameters	Leaf Area Index $M_{\text{avg}}^{\text{LAI}}$	Number of Leaves $M_{\text{avg}}^{\text{Leaf}}$	Cumulative Amount of Light $M_{\text{avg}}^{\text{Flux}}$ ($\mu \text{mol}\cdot m^{-2}\cdot s^{-1}$)
Growth Stage				
Seedling stage		[0.8, 1.7)	[1,3)	[0,126220)
Growth stage		[1.7, 3.3)	[3,9)	[126220, 320000)
Harvest stage		[3.3, 5)	[9,12)	[320000, ∞)

Table 4 shows the environmental conditions of temperature and humidity range corresponding to various growth stages, where $T_{o}^{n}$ and $R_{o}^{n}$ represent the preset temperature and humidity of the Zone #*n*, respectively. The setup range in terms of temperature and humidity is based on the acquired parameters of previous cultivation experiments. These parameters are suitable for the current cultivation environment. When the cultivation site differs, the interior parameter still requires correction.

**Table 4:** Target temperature and humidity range corresponding to various growth stages.

	Stage	Seedling Stage	Growth Stage	Harvest Stage
Range				
Temperature $\left[T_{\min}^{n,v},\;T_{\max}^{n,v}\right]$		$\left[T_{o}^{n}-1\text{, }T_{o}^{n}+1\right]$	$\left[T_{o}^{n}+1\text{, }T_{o}^{n}+2.5\right]$	$\left[T_{o}^{n}-3\text{, }T_{o}^{n}+1\right]$
Humidity $\left[R_{\min}^{n,v},\;R_{\max}^{n,v}\right]$		$\left[R_{o}^{n}-8\text{, }R_{o}^{n}\right]$	$\left[R_{o}^{n}-10\text{, }R_{o}^{n}-5\right]$	$\left[R_{o}^{n}\text{, }R_{o}^{n}+20\right]$

#### 2.2.4. Set-Points Module

This module can randomly set the temperature and humidity expectations according to the temperature range and humidity range values of the current stage of the plants (in automatic control mode). The user can manually set the expected temperature and humidity values of various culture areas.

## 3. System Implementation

**Figure 4:**
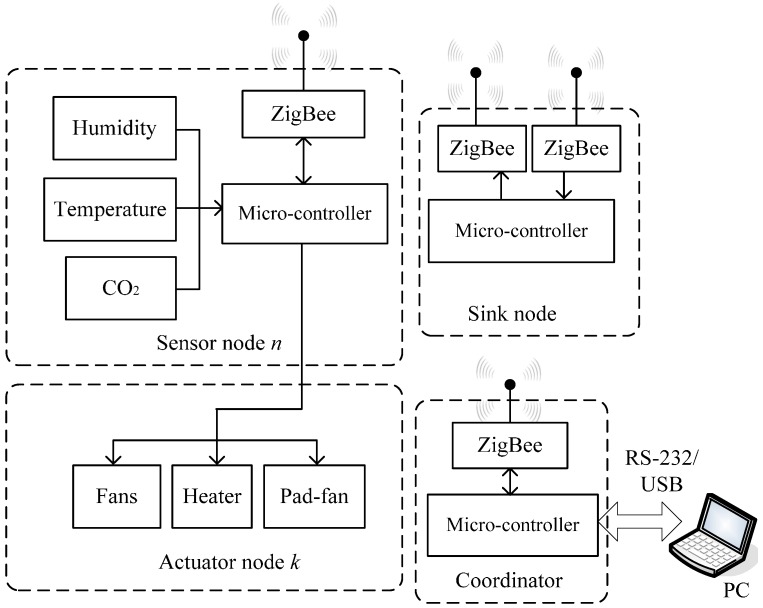
System structure with wireless sensor network.

Figure 4 shows the software and hardware architecture of the overall system, including the sensing and communication module, actuator module, control module, and PC. The controller at each sensor node receives the environmental parameter data of the node via the wireless ZigBee module, including temperature and humidity sensors, the $CO_{2}$ sensor, and the light quantum sensor. These values are sent by the ZigBee module on the controller of the node to the controller at the coordinator node. This controller integrates the sensor data of the various nodes and sends them to a local side controller. The local side controller transfers the data to the computer to instantly display the environmental data of various sensor nodes, and the output values of the actuators in various zones are decided by fuzzy logic control according to the feedback values. Finally, the output values are transferred to the controller of the actuator node in various zones, and the controller drives the heater, fans, and humidifier for environmental control. The hardware design implementation and software development process are introduced, as follows.

### 3.1. Hardware

#### 3.1.1. Sensor Node

**Figure 5:**
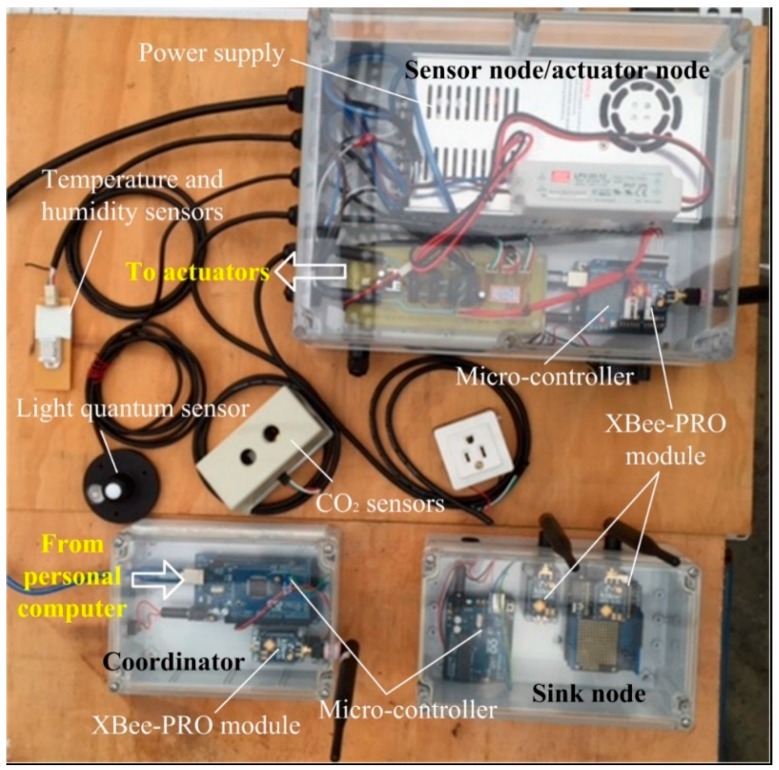
The MEM-based components in the system.

In this system, each sensor node is embedded with a temperature and humidity sensor, a $CO_{2}$ sensor, and a luminance sensor. The model of the temperature and humidity sensor is DHT22 (Model: AM2303) [38], the model of the CO_2_ sensor is MH-Z14 [39], the model of the luminance sensor is Lightscout 3668I [40], and the service voltage is 5 V. The output end of these sensors is connected to the controller input/output port, and the collected data are sent by the ZigBee communication module on the controller (model: XBee-PRO^®^ 900 HP [41]) to the coordinator. XBee-PRO 900 HP embedded modules offer best-in-class range wireless connectivity to devices. This module utilizes the featuring dense network operation and support for sleeping routers and DigiMesh^®^ networking protocol. They also provide support to a proprietary point-to-multipoint configuration. Figure 5 demonstrates the MEM-based components in the system. The sensor node and actuator node share one micro-controller. The sink node includes two ZigBee modules and one micro-controller. The coordinator is made up of one micro-controller and one ZigBee module.

#### 3.1.2. Actuator Node

The actuators in the actuator node include a humidifier (with a set of fans), a heater (with a set of fans), and a circulating fan. The humidifier is a pad-fan (with two sets of fans) for humidification. The direct-current (DC) fan blows the drip into the environment, humidifying and cooling the regional environment. The microcontroller sends a pulse-wide-modulation (PWM) signal to the solid-state-relay (SSR) to control the pad-fan output. The heater consists of a set of heating cords and two sets of fans. One set of fans draws air into the heater, the heat energy is emitted when the heating cord is short circuited, and then the heat is removed by the other set of fans. In this heating process, though heating is fast, the box will not be overheated, so there is no overheated air blown out, which would injure the plants. The control mode of the heater is the same as the humidifier, meaning the PWM signal drives the on/off of SSR relay to control the output. The circulating fan control circuit also uses the SSR relay to switch the fan on and off. In addition, a set of $CO_{2}$ supply devices is placed in the greenhouse, which supplements the amount of $CO_{2}$, in order to have the $CO_{2}$ concentration reach the expected level.

#### 3.1.3. Control Node

The sensor/actuator nodes in each cultivation zone are embedded with a micro-controller, and the coordinator and sink node have a set of controllers. The appropriate controller is selected according to the data size to be processed and the memory space. The Arduino-MEGA development board is utilized in the sensor/actuator node and coordinator. The Arduino-UNO development board is utilized in the sink node.

### 3.2. Software

#### 3.2.1. Graphic User Interface

**Figure 6:**
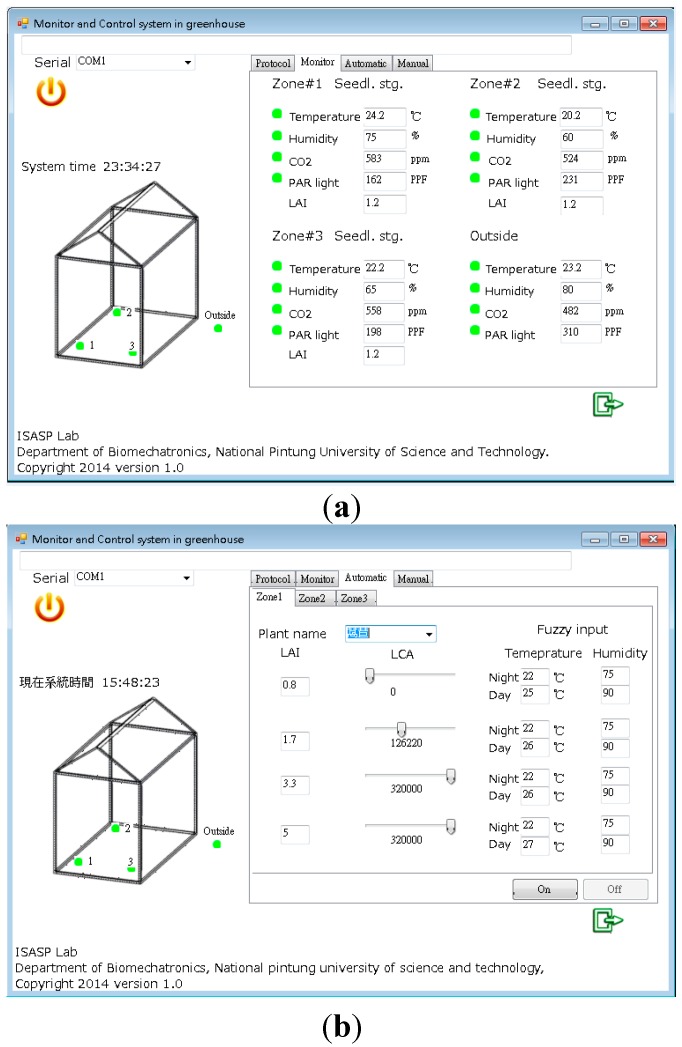
Monitoring of environmental parameters of various zones. (**a**) Monitoring of environmental parameters of various zones; (**b**) parameters setting under automatic control mode.

This study uses Visual Basic software to design the human-machine interface, which is designed with four window menus. The first window displays the transmission protocol setting of the communication transmission port. The second part displays the present environmental parameter variable values of all zones (see Figure 6a). The third part is the window menu of the parameters to be preset in the fuzzy control mode (see Figure 6b). The level of photo flux determines the day and night. The fourth part is the menu for the manual control mode. The automatic control mode setting window is displayed by clicking “Automatic”. The window displays “Zone1”, “Zone2”, and Zone3 menus, which correspond to three planting zones. The user can set the threshold of cumulative light and leaf area values, as well as the corresponding temperature and humidity values for various plant growth stages. Finally, in the window menu of manual control, if the user clicks “Manual”, the window displays the on/off state of the actuating devices in various culture areas. It is noteworthy that when the system is in automatic control mode, the user cannot perform any operations in manual mode.

**Figure 7:**
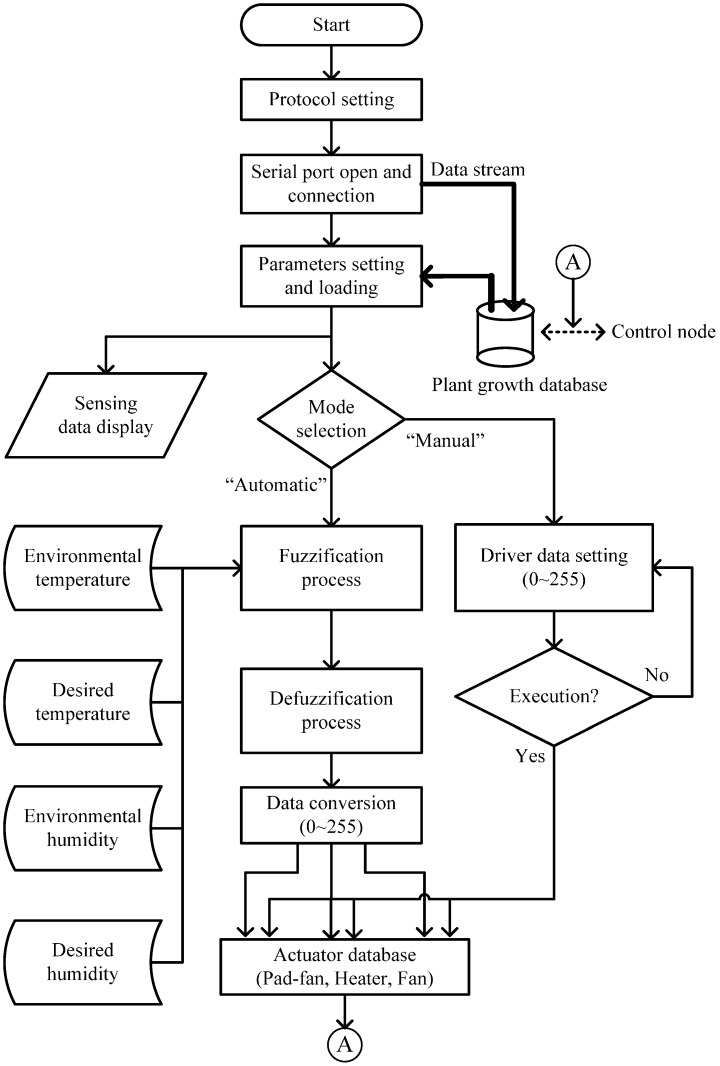
Flowchart of GUI program design.

The flowchart of manual and automatic operation program is shown in Figure 7. The system will initially receive sensor data from the control module and present it in a GUI. The fuzzification and defuzzification program is written by VB software based on Equations (1)–(11). The program has a built-in 9-rule database for the system to perform decision functions. When the system completes the defuzzification process, the three normalized control data are generated. The data are converted to 0~255 PWM driver signals to each actuator through a data conversion process. In addition, the system also drives the actuators through manual operation.

#### 3.2.2. Data Reception and Control Program

The data acquisition, synchronization and reception of sensor data is decided by the Arduino micro-controller in the coordinator. The ZigBee module is employed to achieve the functions of data transmission and reception. The work flow program of the control module is depicted as follows.

*Define variables*
**Input variable**: sensor data (Temperature, Humidity, CO2, and Light intensity);
**Output variable**: actuator data (Heater, Pad-fan, and Fans);
**Communication:** protocol setup and port connection (PC and ZigBee);
*Begin*
  **Loop 1**
    **{** Receive the actuator data from PC;
 Remove wrong data. 
      Decode the actuator data;
      Send the decoded actuator data to actuator via Zigbee;
 **}** 
  **Loop 2**	
    **{** Receive the sensor data via Zigbee;	
 Remove wrong data. 
      Decode sensor data;
      Send the decoded sensor data to PC;
    **}**
*End*



The above process shows that the control module will decode each actuator datum sent from the PC. When the received number of data reaches the target number, the collected data is then sent from ZigBee to the actuator. This process is repeated. The PC keeps sending actuator data to the control node every five seconds. On the other hand, the controller also decodes each sensor data transmitted from the sensor. When the number reaches the target number, these data are sent to PC. It is noteworthy that the interval of data transmission for each sensor differs. The longest interval is adopted to acquire sensor data so that all the sensor data is collected prior to the data transmission process.

### 3.3. System Integration and Testing

After the overall systems are designed and completed, the execution of software and hardware modules is tested. According to the test results, the temperature deviation and humidity deviation detected by the DHT22 and commercial recorder (Model: TR-74Ui [42]), respectively, is ±0.4 °C at 65% RH and ±2% at 27 °C. The result demonstrates the stability is sufficient for an outdoor experiment. The concentration range detected by the $CO_{2}$ sensor (MH-Z14) is 0 ppm–5000 ppm, the warm-up period is about 4 min, and then the $CO_{2}$ concentration value becomes stable. The XBee-PRO^®^ 900HP module with 2.1 dB dipole antennas is tested in an unshaded outdoor environment, and data reception within 6 km is normal; however, if there is a medium, such as a glass window or cement wall, between the receiving and transmitting modules, the transmission distance decreases to about 290–305 m. In addition, to test the communication’s multiplexing scope, the ZigBee module is dispersed and deployed with the spacing of 150 m between each other. The distance between each ZigBee module and the reception module is 300 m. The experiment results show that its communication distance can reach 200 m and remain stable and robust among crowded buildings. In the end, the results matched expectations.

**Figure 8:**
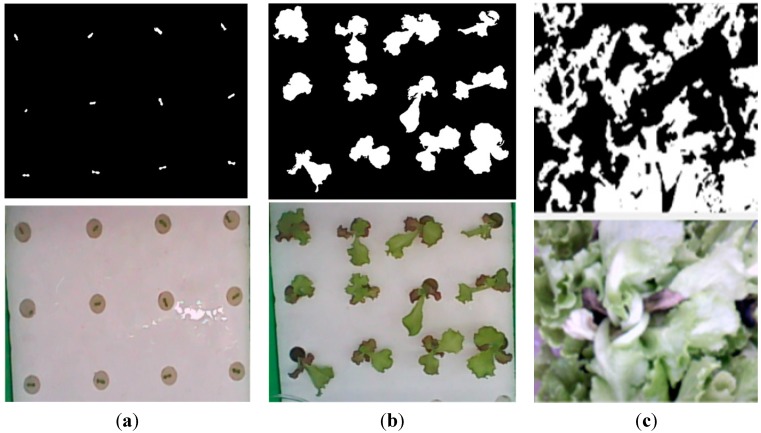
The results of the image process. (**a**) Seedling stage; (**b**) growth stage; (**c**) harvest stage.

Some literature discusses transmission energy attenuation of the ZigBee module and incorrect reception data, and proposes some mathematic models to depict such a phenomenon [30,43]. This study does not take into account the abovementioned factors, which cause data loss during transmission or sensor anomalies. However, even with the above problems, the unreliable data can be removed through post-processing of recorded data offline. In terms of the heater test, when the confined space is 0.48 m^3^, in a full voltage test, the heating rate is 2.1 °C/min; in the same volume and full voltage supply, the humidification rate of humidifier is 5%/min. When the fan is supplied with full voltage, the maximum air velocity is 2.1 m/s. In terms of software testing, the information sent from the control node to the PC is a continuous value, as initiated by the letter “S” to transmit instructions, and then the environmental sensing values of various nodes, in the human-machine interface, the values are segmented and stored in a matrix, and then the data in the matrix are read according to the user requirement. Besides, the NI myRIO device [44] is used to acquire the image of plant leaves and calculate the LAI and number of leaves through NI LabVIEW interface. After calculation, the data is received by the GUI program and sent to the growth stage selector to determine the current growth stage of the plant. Figure 8 demonstrate the image process results for leaf area detection under different plant growth stages.

## 4. Experimental Results and Discussion

### 4.1. Culture Conditions

The target crop in this study was leaf lettuce, as provided by the Known You company. The hydroponics are utilized for lettuce cultivation and the sponge foam was served as growth medium. Styrofoam plates were used to shield nutrient solution from light, and to avoid lichen breeding in the nutrient solution. There are 18 hollow pots in the plate for fixing the sponge foam. Each pot was sown with three seeds secured with foam, which were thinned to one plant per pot after 4 day. The nutrient solution was changed every 10 days, and was prepared by mixing 1.8 g ammonium nitrogen, 8.7 g carbon nitrogen nitrate, 9 g water soluble phosphoric anhydride, and 28.5 g water soluble potassium oxide, 48 g in all, with 1 L of water. The bubbler is started every three hours, and works for 15 min each time, in order to maintain the dissolved oxygen in the nutrient solution. The electrical conductivity of the nutrient solution is maintained at 1.1~1.3 ms, the pH is maintained at 6~6.5.

### 4.2. Experimental Setup

**Figure 9:**
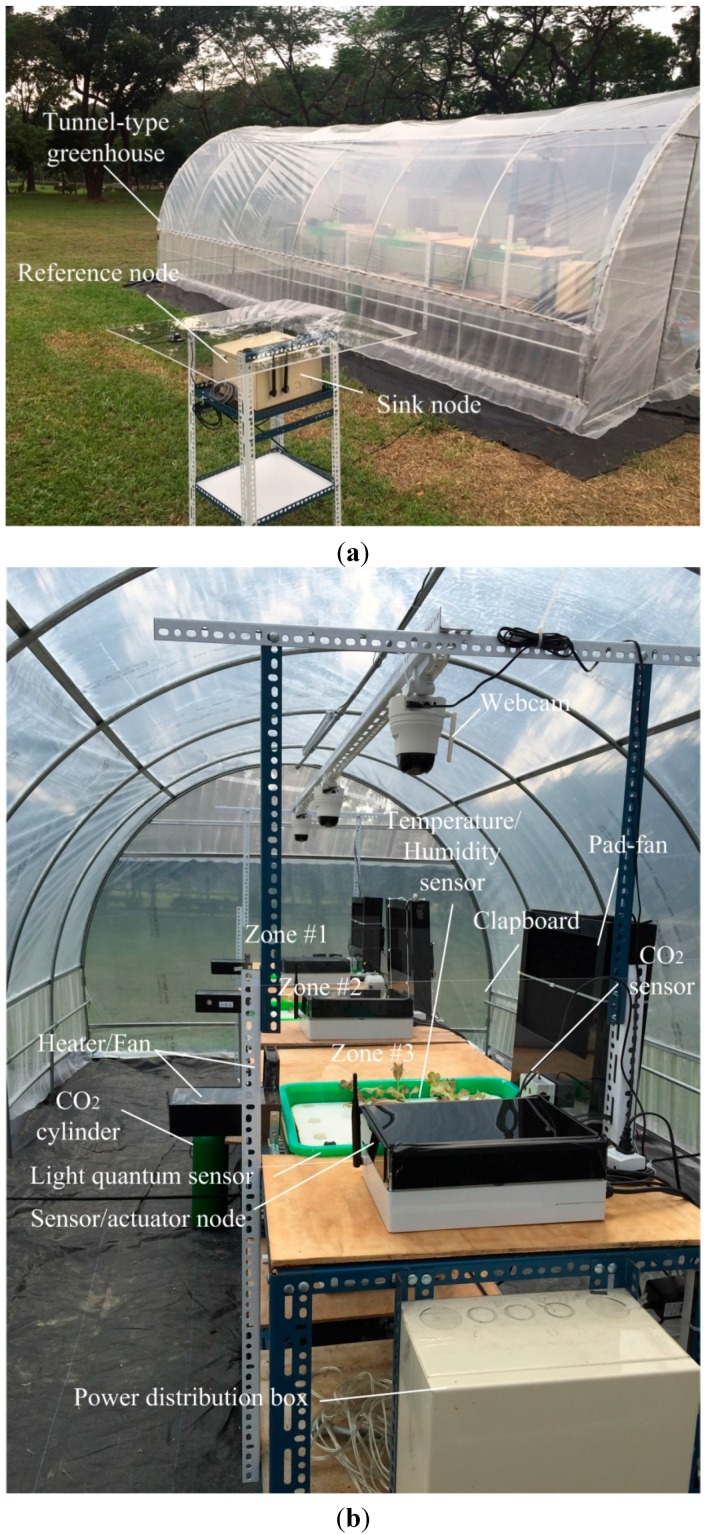
The configuration of plant cultivation zones in experimental site. (**a**) Appearance of tunnel-type greenhouse; (**b**) snapshot of experimental site.

Three different cultivation scenarios are used to validate and analyze the performance of proposed system. The first experiment aims to test whether the system can maintain consistent environmental conditions of various zones. The second experiment aims to test whether the system can control the temperature and humidity of various zones at different levels. The above experiments disregard the growth conditions of plants. The target of the temperature and humidity of various zones are preset in order to observe whether the proposed system can reach the target values. The third experiment is to regulate the temperature and humidity considering the plant growth response. As limited to the heating, air supply, and humidifying capacities of the actuator module, a tunnel-type greenhouse (480-cm × 240-cm × 270-cm (Width (W) × Length (L) × Height (H)) is utilized for lettuce planting, in order to validate and analyze the performance of proposed system in regulating the parameters of various zones. Figure 9 shows the appearance of the experimental site. The greenhouse is enclosed by transparent material and contains three planting zones, and each zone is 120-cm × 110-cm (L × W). There are baffles between the culture areas. The baffle is 50-cm × 110-cm (H × W). There are sensing and communication modules, actuator modules, and a control module in the planting zones, and there are sensing and communication modules (reference node) outside the greenhouse for monitoring the temperature and humidity changes outside the greenhouse. The CO_2_ replenishing device is placed in the lower part of the second culture area (Zone #2), and CO_2_ is conveyed by taps to various planting zones, in order that the CO_2_ concentration is uniformly distribution in various zones. There is a webcam above each zone for recognizing the plant growth stage. There are three sets of fans, which are the circulating fan, the fan for the heater, and the fan for the humidifier, which are placed in the corners of each zone for creating air guide walls around the planting area. This configuration of modules forms a microenvironment climatic region in the planting zones.

a. Scenario 1

**Figure 10:**
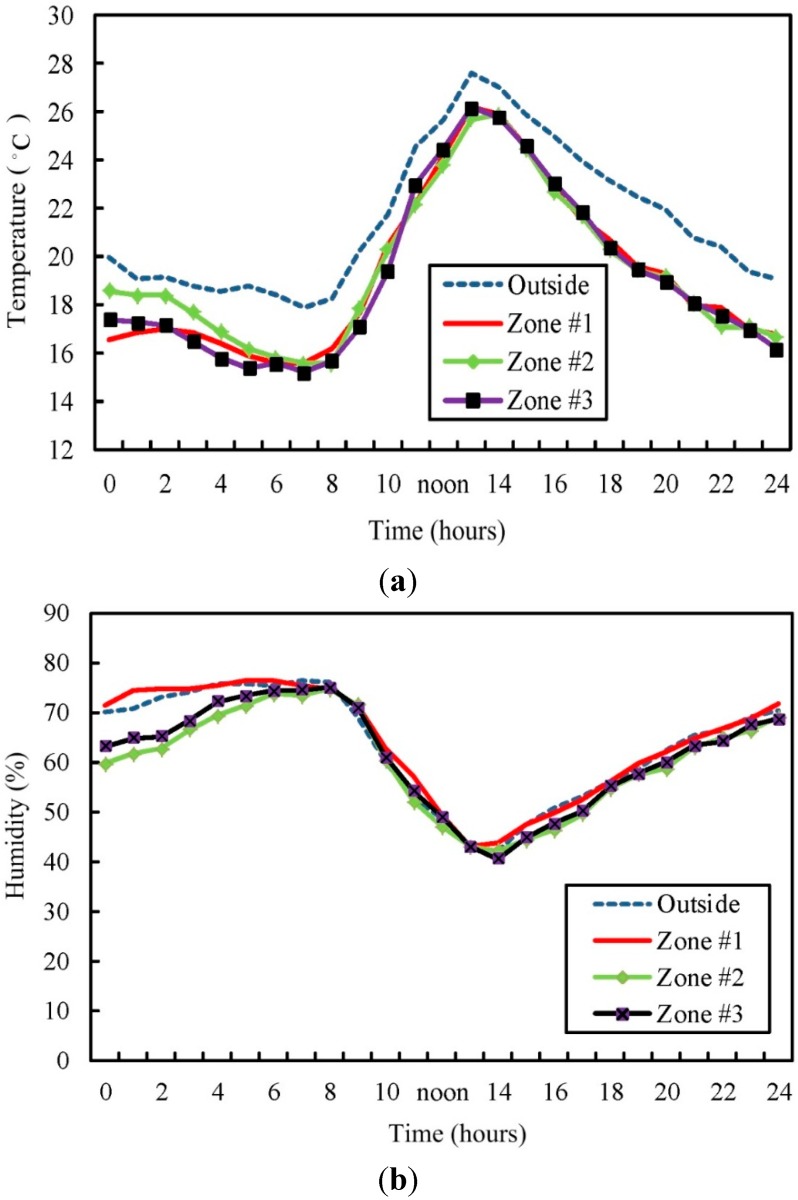
Temperature and humidity curves in various zones and outside greenhouse (Scenario 1). (**a**) Temperature curve; (**b**) humidity curve.

In this experiment, the expected temperature in the three cultivation zones is set to 3 °C lower than the outside temperature, and the humidity is identical with that outside the greenhouse. The initial temperature and humidity of the various zones are different, and then this system is actuated and the ambient temperature and humidity changes in various zones are observed for one day. Figure 10 shows the temperature and humidity changes in various zones. It is observed that when the outside temperature gradually increases, the difference between the temperatures inside and outside the greenhouse controlled by the system gradually decreases to 1.2 °C. That is, it is difficult to maintain a temperature difference of 3 °C between the inside and outside of a greenhouse in terms of temperature control. When the outside temperature decreases to 20 °C, the system can control the difference between the temperatures inside and outside the greenhouse within 2.6 °C. In terms of humidity control, the humidity deviation is about 2.5%.

b. Scenario 2

In this experiment, the initial environment parameters of each zone are consistent, and the target range of temperature and humidity are manually set, thus creating different temperature and humidity conditions in each zone. The environment parameters in each zone are set as follows:

The preset temperature and humidity is set as $T_{0}^{1}=$ 21 °C and $R_{0}^{1}=$ 80% for Zone #1, respectively. The target temperature and humidity for Zone #1 range between 20 °C and 22 °C, respectively. Similarly, the parameter setting for Zone #2 and Zone #3 is shown in the following Table 5 accordingly.

**Table 5:** The parameter setting in each zone for scenario 1.

	Stage	Zone #1	Zone #2	Zone #3
Parameters				
Temperature	Preset ($T_{0}^{n}$)	21.2 °C	19.5 °C	23 °C
	Target ($T_{set}^{n}$)	21 °C	19 °C	23 °C
	Range $\left[T_{\min}^{n,v},\;T_{\max}^{n,v}\right]$	[20, 22]	[18, 20]	[22, 24]
Humidity	Preset ($R_{0}^{n}$)	80%	70%	75%
	Target ($R_{set}^{n}$)	75%	65%	70%
	Range $\left[R_{\min}^{n,v},\;R_{\max}^{n,v}\right]$	[72, 80]	[62, 70]	[67, 75]

**Figure 11:**
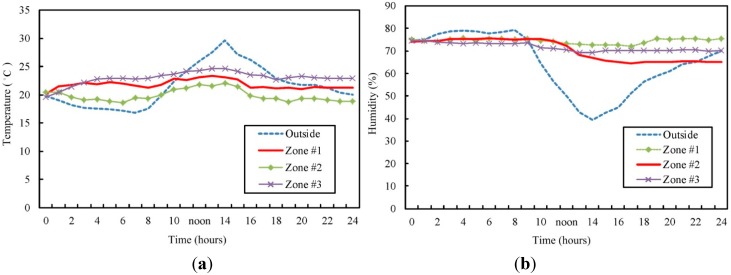
Comparisons of diurnal changes of parameters in each zone; (**a**) temperature; (**b**) humidity.

Whether the zones can be maintained at the range of temperature and humidity is observed under the regulation and control of the proposed system. The parameters are recorded once every 1 h, the mean of which is obtained after the duration of 24 h. Figure 11 shows the changes in the temperature and humidity in each zone. The average temperature of Zone #1 is 21.8 °C, humidity 75.1%; the average temperature of Zone #2 is 19.8 °C, humidity 65.6%; the average temperature of Zone #3 is 22.6 °C, humidity 69.2%. The result shows that at noon (12:00–14:00), the higher temperature outside the greenhouse results in an increase of average temperature inside the greenhouse. However, the temperature stays within the desired range and the humidity remains the same.

c. Scenario 3

**Figure 12:**
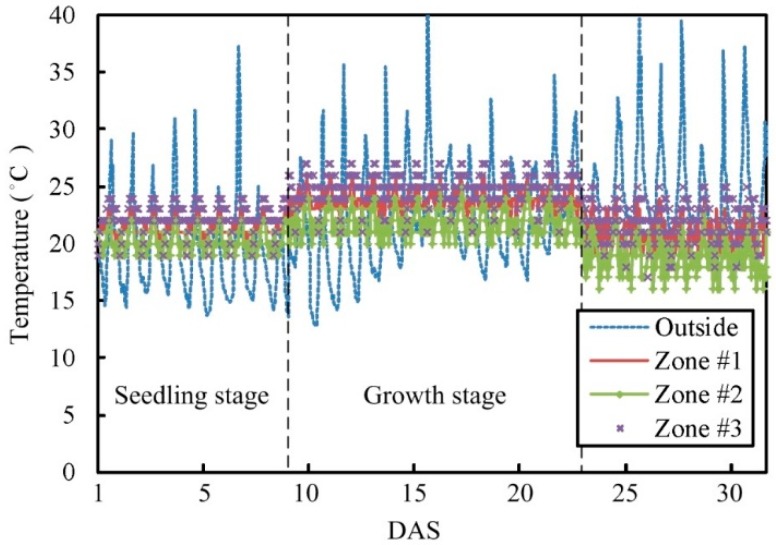
Comparisons of temperature parameter changes between Zone #1 to Zone #3 (under automatic mode).

**Figure 13:**
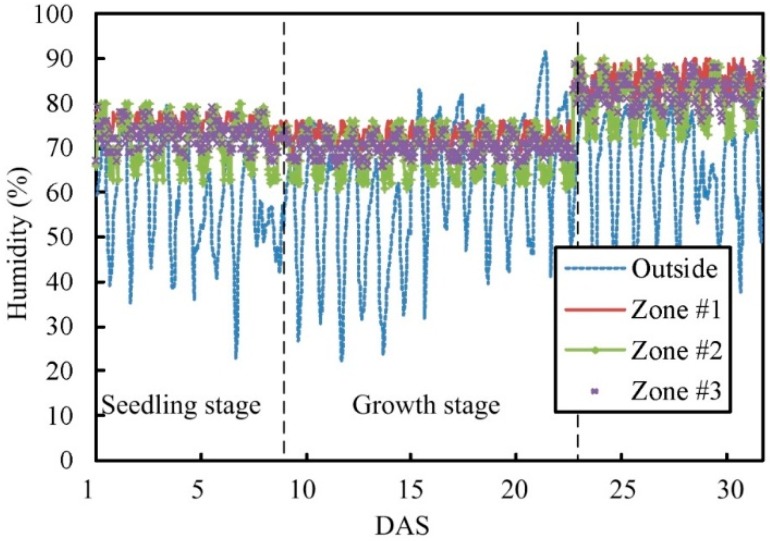
Comparisons of humidity parameter changes between Zone #1 to Zone #3 (under automatic mode).

In this experiment, the lettuce is cultivated in Zone #1, Zone #2, Zone #3, and the uncontrolled zone within the greenhouse. The initial environment parameters of each zone are different. The crop harvest was performed at 30 days after sowing (DAS). In this cultivation experiment, the temperature and humidity values are regulated based on the growth response of plant. The preset temperature and humidity is set as $T_{0}^{n}=$ 20 °C and $R_{0}^{n}=$ 80% for each zone (*n* = 1, 2, 3), respectively. The average CO_2_ concentration is 600 ± 75 ppm and the average photo flux is between 85 and 210 $\mu \text{mol}\cdot m^{-2}\cdot s^{-1}$ in the greenhouse. This is equivalent to the average daily light integral (DLI) from 3.6 to 9.07 $\text{mol}\cdot d^{-1}$. Figure 12 and Figure 13 show the changes in the temperature and humidity during plant growth, and show the changes of temperature and humidity outside the greenhouse, where the dotted line represents the switching time point of the plant growth stage. During 9 DAS to 10 DAS, the lettuce cultivated in various zones turned from the seedling stage into the growth stage. During day 22 to day 23, the lettuce in various zones has gone from the growth stage to the harvest stage. Due to the change of temperature outside the greenhouse, the temperature inside the greenhouse changes as well. However, the temperature change is still within the desired control range.

**Figure 14:**
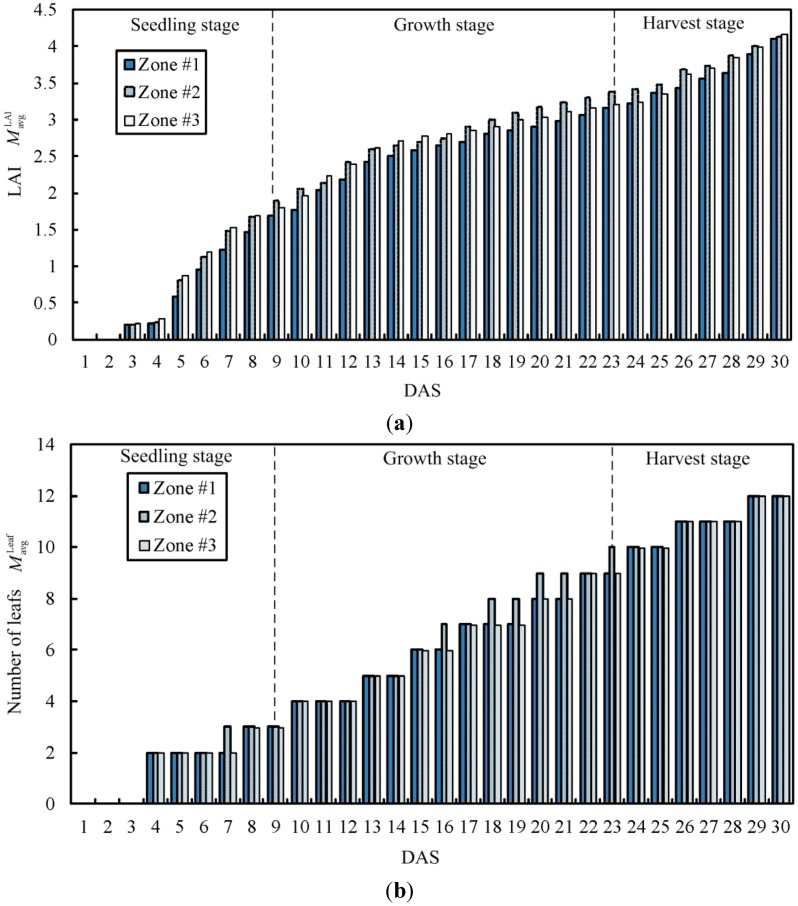

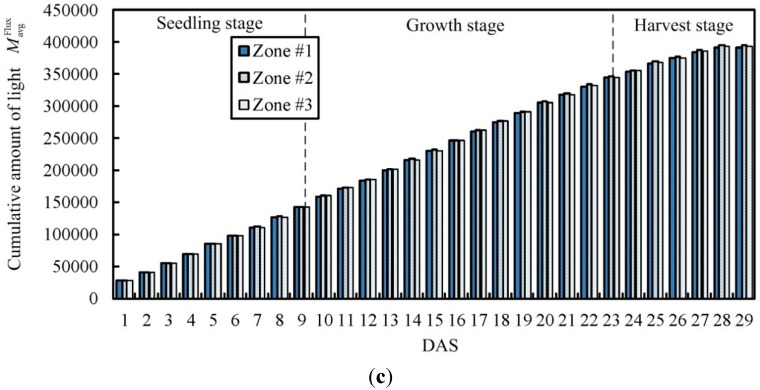
Variations in plant growth patterns: (**a**) LAI *versus* DAS; (**b**) number of leaves *versus* DAS; (**c**) cumulative amount of light *versus* DAS.

Figure 14 indicates the changes in the daily LAI of lettuce, number of leaves and cumulative amount of light. As a complete knowledge database for the relations between the growth response and the plant growth conditions had already been established, the output of the “data transformer” could be compared with the growth response of the corresponding plants in the database. The plant growth information in the database can serve as a reference for decentralized control required by the proposed system. The main measurements of harvested crops taken were average fresh mass, dry mass, number of leaves, and height of lettuce, shown in Table 6. The data shows that all treatment results for lettuce cultivation in each zone are not significantly different statistically. Relatively, the result of growth response of plant in uncontrolled area is different from that in other zones. It is indicated that the proposed system can control some plant growth conditions in terms of temperature and humidity in each zone for standardized planting.

**Table 6:** The tissue analysis of Lettuce under different growth conditions.

Parameters	Zone #1	Zone #2	Zone #3	Uncontrolled Zone
Height of plant (cm)	24.9 ± 0.9	24.4 ± 1.4	24.1 ± 1.1	23.1 ± 0.8
Number of leaves	12 ± 1	12 ± 1	12 ± 1	11 ± 1
Shoot dry mass (g/plant)	3.8 ± 0.2	3.8 ± 0.6	3.8 ± 0.3	3.2 ± 0.5
Shoot fresh mass (g/plant)	30.1 ± 1.3	30.2 ± 1.5	30.2 ± 1.2	24.2 ± 1.5

### 4.3. Discussion

According to above experimental results, when the system is in automatic mode, the temperature and humidity parameters in various zones can be regulated based on the target range of temperature and humidity at the lettuce growth stage. The proposed scheme allows the user to cultivate the same or different kinds of crops in the same greenhouse. It offers the most suitable growth environment based on the growth conditions of crops. In contrast to the common plant growth facilities, this system is more flexible and more suitable for cultivating a small area with different crops. Thus, there is no need to use multiple greenhouses to cultivate different crops, which greatly saves on costs and space. Besides, in order to maintain a consistent environment for the benefit of plant growth, the common plant growth facilities needs to consume large amounts of energy for cultivation. With regards to the proposed system, the indoor environment parameters are adjusted real-time based on the plant growth response, temperature and humidity outside the greenhouse. Doing so can reduce energy consumption from using plant growth facilities. Although this system is limited by its failure to adjust the temperature and humidity of a large range, its benefits in terms of energy consumption meet the demand with regard to cropping suitability. However, this system uses a small heater, pad-fan, and fans, and the effects of heating, cooling, humidification, and dehumidification are limited to a space of a specific size. If it is used in large greenhouses in the future, additional cooling equipment, blowers, or a large wetted pad system can be installed for environmental control in a large greenhouse.

## 5. Conclusions

In this paper, a prototype for a proposed scheme based on a fuzzy rule-based system has been proposed, and it is used to control and monitor environmental parameters in various zones within a greenhouse. The prototype of the proposed system has been preliminarily implemented in an outdoor tunnel-type greenhouse. The experimental results show that the proposed system can effectively regulate the inside temperature and humidity according to plant growth response in each zone. When the environmental parameters in each zone of the greenhouse are inconsistent due to uncertainties, the proposed system can adjust the parameter to keep it consistent in each zone. In this way, the growth quality of plants is consistent. This method can be applied in each zone of the greenhouse to intentionally adjust the environment parameters of plant growth to achieve diversified cultivation.

Furthermore, users can observe the growth response of plants and monitor environmental factors within the cultivation zone remotely at any time in order to effectively enhance the quality and quantity of plant production. All sensors and controllers are made by means of MEM technology, and these high-stability modules can be duplicated based on user demand. It is low in terms of hardware implementation costs and flexible in terms of hardware modification.

In the future, this proposed technique will be combined with the supplemental lighting system and the cloud-based plant production platform to monitor plant growth, improve the growth quality of plants and enhance quantity of plant production [45].

## References

[1] Ottoson J.R., Nyberg K., Lindqvist R., Albihn A. (2011). Quantitative microbial risk assessment for Escherichia coli O157 on lettuce, based on survival data from controlled studies in a climate chamber. J. Food Protect..

[2] Webb S.E., Hochmuth R.C. Vegetable Insect Identification and Management-Florida Greenhouse Vegetable Production Handbook. https://edis.ifas.ufl.edu/cv274.

[3] Holvoet K., Sampers I., Seynnaeve M., Jacxsens L., Uyttendaele M. (2015). Agricultural and management practices and bacterial contamination in greenhouse *versus* open field lettuce production. Int. J. Environ. Res. Public Health.

[4] Heidemann J., Govindan R., Levine B., Hristu D. (2005). Embedded Sensor Networks. Handbook of Networked and Embedded Control Systems.

[5] Zhang Q., Yang X.L., Zhou Y.M., Wang L.R., Guo X.S. (2007). A wireless solution for greenhouse monitoring and control system based on ZigBee technology. J. Zhejiang Univ. Sci. A.

[6] Candido A., Cicirelli F., Furfaro A., Nigro L. (2007). Embedded real-time system for climate control in a complex greenhouse. Int. Agrophys..

[7] Yunseop K., Evans R.G., Iversen W.M. (2008). Remote sensing and control of an irrigation system using a distributed wireless sensor network. IEEE Trans. Instrum. Meas..

[8] Speetjens S.L., Janssen H.J.J., van Straten G., Gieling T.H., Stigter J.D. (2008). Methodic design of a measurement and control system for climate control in horticulture. Comput. Electron. Agric..

[9] Pawlowski A., Guzman J.L., Rodriguez F., Berenguel M., Sanchez J., Dormido S. (2009). Simulation of greenhouse climate monitoring and control with wireless sensor network and event-based control. Sensors.

[10] Park D.H., Park J.W. (2011). Wireless sensor network-based greenhouse environment monitoring and automatic control system for dew condensation prevention. Sensors.

[11] Coates R.W., Delwiche M.J., Broad A., Holler M. (2013). Wireless sensor network with irrigation valve control. Comput. Electron. Agric..

[12] Ahonen T., Virrankoski R., Elmusrati M. Greenhouse monitoring with wireless sensor network. Proceedings of the IEEE/ASME International Conference on Mechatronic and Embedded Systems and Applications.

[13] Fitch M., Nekovee M., Kawade S., Briggs K., MacKenzie R. (2011). Wireless services provision in TV white space with cognitive radio technology: A telecom operators perspective and experience. IEEE Commun..

[14] Chang C.L., Hong G.F., Li Y.L. (2014). A supplementary lighting and regulatory scheme using a multi-wavelength LED module for greenhouse application. Light. Res. Technol..

[15] Paek J., Hicks J., Coe S., Govindan R. (2014). Image-based environmental monitoring sensor application using an embedded wireless sensor network. Sensors.

[16] Ferreira P.M., Fariab E.A., Ruano A.E. (2002). Neural network models in greenhouse air temperature prediction. Neuro Comput..

[17] El Ghoumari M.Y., Tantau H.J., Serrano J. (2005). Non-linear constrained MPC: Real-time implementation of greenhouse air temperature control. Comput. Electron. Agric..

[18] Teitel M., Atias M., Barak M. (2010). Gradients of temperature, humidity and CO_2_ along a fan ventilated greenhouse. Biosyst. Eng..

[19] Fitz-Rodriguez E., Kubota C., Giacomelli G.A., Tignor M.E., Wilson S.B. (2010). Dynamic modeling and simulation of greenhouse environments under several scenarios: A web-based application. Comput. Electron. Agric..

[20] Park D.H., Kang B.J., Cho K.R. (2011). A study on greenhouse automatic control system based on wireless sensor network. Wirel. Pers. Commun..

[21] Chen F., Tang Y.N., Shen M.Y. (2011). Coordination control of greenhouse environmental factor. Int. J. Autom. Comput..

[22] Chang C.L., Sie M.F. (2012). A multistaged fuzzy logic scheme in a biobotanic growth regulation system. HortScience.

[23] Chao K., Gates R.S., Sigrimis N.A. (2000). Fuzzy logic controller design for staged heating and ventilating systems. Trans. ASAE.

[24] Wang L.X. (1992). Generating fuzzy rules by learning from examples. IEEE Trans. Syst. Man Cybern..

[25] Herrera F., Lozano M., Verdegay J.L. (1995). Tuning fuzzy logic controllers by genetic algorithms. Int. J. Approx. Reason..

[26] Dai X., Li C.K., Rad A.B. (2005). An approach to tune fuzzy controllers based on reinforcement learning for autonomous vehicle control. IEEE Trans. Intell. Transp. Syst..

[27] Kaelbling L.P., Littman M.L., Moore A.P. (1996). Reinforcement learning: A survey. J. Artif. Intell. Res..

[28] Desouky S.F., Schwartz H.M. (2011). Q(*λ*)-learning adaptive fuzzy logic controllers for pursuit–evasion differential games. Int. J. Adapt. Control Signal Process..

[29] Xing F., Tian Y.C., Li Y., Sung Y. (2007). Wireless sensor/actuator network design for mobile control applications. Sensors.

[30] Lin S., Zhou G., Whitehouse K., Wu Y. Towards stable network performance in wireless sensor networks. Proceedings of the 30th IEEE Real-Time Systems Symposium.

[31] Chao K., Gates R.S. (1996). Design of switching control systems for ventilated greenhouses. Trans. ASAE.

[32] Lees M.J., Taylor J., Chotai A., Young P.C., Chalabi Z.S. (1996). Design and implementation of a proportional-integral-plus (PIP) control system for temperature, humidity and carbon dioxide in a glasshouse. Acta Hort..

[33] Marks R.J. (1994). Fuzzy logic technology and applications.

[34] Andriolo J.L., da Luz G.L., Witter M.H., Godoi1 R.D.S., Barros G.T., Bortolotto O.C. (2005). Growth and yield of lettuce plants under salinity. Hortic. Bras..

[35] Linker R., Gutman P.O., Seginer I. (1999). Robust controllers for simultaneous control of temperature and CO_2_ concentration in greenhouses. Control Eng. Pract..

[36] Korner O. (2004). Evaluation of crop photosynthesis models for dynamic climate control. Acta Hort..

[37] Korner O., Aaslyng J.M., Andreassen A.U., Holst N. (2007). Microclimate prediction for dynamic greenhouse climate control. HortScience.

[38] Aosong (Guangzhou) Electronics Co., Ltd AM2303 Datasheet, 2014. https://www.adafruit.com/datasheets/DHT22.pdf.

[39] Zhengzhou Winsen Electronics Technology CO., Ltd MH-Z14 CO2 Module Datasheet, 2013. http://www.futurlec.com/Datasheet/Sensor/MH-Z14.pdf.

[40] Spectrum Technologies Lightscout 3668I, 2014. http://www.specmeters.com/weather-monitoring/sensors-and-accessories/sensors/light-sensors/lightscout-quantum-light-sensor-3668i/.

[41] XBee-PRO^®^ 900 HP Datasheet, Digi International Inc.. http://www.digi.com/pdf/ds_xbeepro900hp.pdf.

[42] T&D Corporation TR-74Ui Datasheet, 2012. http://www.tandd.com/product/tr74ui/option.html.

[43] Rassam M.A., Maarof M.A., Zainai A. (2014). Adaptive and online data anomaly detection for wireless sensor system. Knowl.-Based Syst..

[44] National Instruments Corporation NI myRIO Datasheet, 2013. http://digital.ni.com/manuals.nsf/websearch/EE9557A29E63D0F986257BB8005700C5.

[45] Roy Fisher R., Ledwaba L., Hancke G., Carel Kruger C. (2015). Open hardware: A role to play in wireless sensor networks?. Sensors.

